# Growth of late preterm infants fed nutrient-enriched formula to 120 days corrected age—A randomized controlled trial

**DOI:** 10.3389/fped.2023.1146089

**Published:** 2023-05-02

**Authors:** Karen P. Best, Lisa N. Yelland, Carmel T. Collins, Andrew J. McPhee, Geraint B. Rogers, Jocelyn Choo, Robert A. Gibson, Teresa Murguia-Peniche, Jojy Varghese, Timothy R. Cooper, Maria Makrides

**Affiliations:** ^1^ Women and Kids Theme, South Australian Health and Medical Research Institute, Adelaide, SA, Australia; ^2^ Adelaide Medical School, The University of Adelaide, Adelaide, SA, Australia; ^3^ School of Public Health, The University of Adelaide, Adelaide, SA, Australia; ^4^ Lifelong Health Theme, South Australian Health and Medical Research Institute, Adelaide, SA, Australia; ^5^ Infection and Immunity, Flinders Health and Medical Research Institute, College of Medicine and Public Health, Flinders University, Bedford Park, SA, Australia; ^6^ School of Agriculture, Food and Wine, University of Adelaide, Adelaide, SA, Australia; ^7^ School of Medicine, Indiana University, Evansville, IN, United States; ^8^ Medical Sciences, Mead Johnson Nutrition|Reckitt, Evansville, IN, United States; ^9^ Department of Neonatology, Lyell McEwin Hospital, Adelaide, SA, Australia

**Keywords:** preterm, nutrition, infant feeding, nutrient enriched formula, infant growth, late preterm

## Abstract

**Objectives:**

We aimed to compare the effects of nutrient-enriched formula with standard term formula on rate of body weight gain of late preterm infants appropriately grown for gestational age.

**Study design:**

A multi-center, randomized, controlled trial. Late preterm infants (34–37 weeks' gestation), with weight appropriate for gestational age (AGA), were randomized to nutrient enriched formula (NEF) with increased calories (22 kcal/30 ml) from protein, added bovine milk fat globule membrane, vitamin D and butyrate or standard term formula 20 kcal/30 ml (STF). Breastfed term infants were enrolled as an observational reference group (BFR). Primary outcome was rate of body weight gain from enrollment to 120 days corrected age (d/CA). Planned sample size was 100 infants per group. Secondary outcomes included body composition, weight, head circumference and length gain, and medically confirmed adverse events to 365 d/CA.

**Results:**

The trial was terminated early due to recruitment challenges and sample size was substantially reduced. 40 infants were randomized to NEF (*n* = 22) and STF (*n* = 18). 39 infants were enrolled in the BFR group. At 120 d/CA there was no evidence of a difference in weight gain between randomized groups (mean difference 1.77 g/day, 95% CI, −1.63 to 5.18, *P* = 0.31). Secondary outcomes showed a significant reduction in risk of infectious illness in the NEF group at 120 d/CA [relative risk 0.37 (95% CI, 0.16–0.85), *P *= 0.02].

**Conclusion:**

We saw no difference in rate of body weight gain between AGA late preterm infants fed NEF compared to STF. Results should be interpreted with caution due to small sample size.

**Clinical Trial Registration:**

The Australia New Zealand Clinical Trials Registry (ACTRN 12618000092291). “mailto:maria.makrides@sahmri.com” maria.makrides@sahmri.com.

## 1. Introduction

Infants born between 34- and 37-weeks' gestation, termed late preterm infants, account for 70%–75% of all preterm births (1, 2). Late preterm infants are often considered functionally full term because of their size and the fact that they are generally clinically stable. They are, however, physiologically and metabolically immature and evidence for disparate outcomes between late preterm infants and their term counterparts is increasing (3). This population are more likely to be underweight and short for their age in early childhood compared to term born children and have poorer neurodevelopment, school performance and a greater need for educational assistance (4–7). There has been an increasing advocacy for improved surveillance and effective interventions for this population to address nutritional deficiencies and avoid overweight (3). Several strategies are utilized for improving nutrient intake of preterm infants prior to hospital discharge including fortification of human breast milk and the use of nutrient-enriched infant formula (8–10), however post-discharge recommendations are lacking (11, 12). There is no question that human breast milk is the optimal source of nutrition for young infants, however many preterm infants receive infant formula as a major source of nutrition in the first few months following hospital discharge, which presents an opportunity for continued nutritional intervention beyond the inpatient period. Recent studies of post-discharge, nutrient-enriched formula have focused on the very preterm and very low birth weight infant (10, 12) and reported benefits include improved growth parameters and associations with improved neurodevelopment at 18 months of age (11). There is however, a lack of evidence in appropriately grown for gestational age (AGA) late preterm infants in need of nutrient enrichment and optimal growth. We hypothesize that a nutrient-enriched post-discharge formula will result in improved outcomes for formula feeding late preterm infants, as seen in those born at earlier gestations. The aim of the present study was to determine if late preterm AGA infants fed a formula enriched with higher protein and vitamin D in addition to nutrients postulated to be beneficial; [bovine milk fat globule membrane (MFGM) (13, 14) and butyrate (15)] results in improved rate of growth post discharge compared with infants fed a standard term formula.

## 2. Materials and methods

This multicenter, randomized, double-blind, trial was approved by the Women's and Children's Health Network Human Research Ethics Committee (HREC/17/WCHN/84) and prospectively registered with the Australia New Zealand Clinical Trials Registry (ACTRN12618000092291).

### 2.1. Participants

Mothers of exclusively formula feeding late preterm infants born 34^+0^ to 36^+6^ weeks' gestation were approached in the Neonatal Unit or Postnatal ward from South Australian Maternity Hospitals including Women's and Children's Hospital, Flinders Medical Centre and Lyell McEwin Hospital or in the community (16). Singleton or twin infant pairs were eligible if they were AGA (birthweight >3rd and <97th percentile) (17), and ≤42 weeks post menstrual age (PMA). Singleton term infants born between 39^+0^- and 40^+6^-weeks' gestation and ≤14 days of age were enrolled in the breastfeeding reference group (BFR) if they were AGA (17) and their mothers' intention was to exclusively breastfeed until their infant was ≥120 days of age. Exclusions in all groups included infants born to mothers with diabetes (pre-existing or gestational); history of severe congenital disease/malformation, metabolic disease, immunocompromised or diagnosed with any other condition likely to interfere with normal growth and development.

### 2.2. Randomization and blinding

Late preterm infants were randomized 1:1 to nutrient enriched formula (NEF) or standard term formula (STF) using a secure web-based randomization service and stratified by infant sex, study center and gestational age (<35 weeks' and 35–36^+6^ weeks' gestation). Allocation followed a computer-generated randomization schedule using randomly permuted blocks of size 4 within strata generated by an independent statistician who was not involved with study participants or data analysis. Twins enrolled into the study were assigned to the same study product, resulting in a partially clustered trial with cluster randomization. A unique study identification number was assigned to each infant together with a product code and one of four colors (blue, violet, red, and green). Study formulas were identical in packaging and labelling and identified by the colored label and product code only. Participants, researchers, and laboratory personnel remained unaware of the group assignments until the data analysis was complete.

### 2.3. Study feeding and study procedures

Powdered study formulas, NEF (22 kcal/30 ml) or STF (20 kcal/30 ml), were manufactured in a licensed facility in accordance with Good Manufacturing Practices (Reckitt|Mead Johnson Nutrition, 2,400 W. Lloyd Expressway, Evansville IN, 47721, USA). The NEF had higher inositol, vitamin D, butyrate, and calories from increased protein per 100 kcal than the STF 2.8 g vs. 2.1 g - (Supplementary Table 1). The ratio of whey to casein proteins in NEF was 80:20 and had enriched whey protein-lipid concentrate (5 g/L, source of bMFGM; Lacprodan® MFGM-10, Arla Foods Ingredients P/S, Denmark). All other nutrients, including calcium and phosphorous are consistent with recommended amounts for preterm infants (18, 19). Infants received blinded study formulas from randomization to 120 days CA when formula cans were returned to the infants were subsequently supplied with unblinded STF until 365 days CA. Infant weight, length and head circumference were measured by research staff trained in anthropometrics at enrollment, 40 weeks PMA and 30, 60, 90, 120, 180, and 365 days' CA. Body composition was measured by air displacement plethysmography using PEAPOD (COSMED USA Inc.) at enrolment and 120 days CA. Dietary intake and recall of fecal characteristics and wind were recorded at 30, 60, 90 and 120 days' CA. Adverse events were collected at each study visit. Serious adverse events were defined as any event during the study period that resulted in death, was life threatening or incapacitating, or required hospitalization. SCORing Atopic Dermatitis (SCORAD) assessment (20) was completed at each in-person study visit and infant fecal samples were collected by parents at enrolment, 60 days, and 120 days' CA using Norgen Stool Nucleic Acid Preservation System (Norgen Biotek, ON, Canada). DNA was extracted from fecal samples and microbiome profiling using 16S rRNA gene sequencing (South Australian Genomics Centre, Australia) was performed according to established methods (21). At 120 days' CA, whole blood was collected from infants *via* heel stick or venipuncture on to dried blood spot cards for analysis of vitamin D and butyrate according to established methods (22, 23).

### 2.4. Outcome assessments

The primary outcome was rate of body weight gain from randomization to 120 days' CA. Secondary outcomes included rate of body length and head circumference gain from enrollment to 120 days' CA and indicators of formula tolerance. All groups were compared for growth outcomes to 365 days, body composition, SCORAD, Vitamin D and butyrate, fecal microbiota composition, and adverse events.

### 2.5. Sample size and statistical analysis

The sample size required to detect a 3 g per day difference in weight gain from randomization to 120 days' CA was 200 late preterm infants (100 in each randomized group) based on 80% power, two-sided alpha = 0.05, SD 6 g/day, 10% loss to follow-up and allowing for clustering due to twins (24). The planned sample size for the term breastfeeding reference group was 100 infants. Recruitment difficulties resulted in early termination of the trial which meant our sample size was substantially reduced. The planned statistical methods were modified based on reduced numbers, and hence may differ from the methods specified in the original trial protocol. The primary outcome of rate of body weight gain was compared between treatment groups with adjustment for infant sex using linear regression and generalized estimating equations to account for clustering due to twins. The primary analysis was performed on the available data according to treatment group assignment (intention-to-treat), followed by a secondary per-protocol analysis. Further details and analysis methods for secondary outcomes can be found in the pre-specified statistical analysis plan (see Supplementary Table 2).

## 3. Results

**Figure 1:**
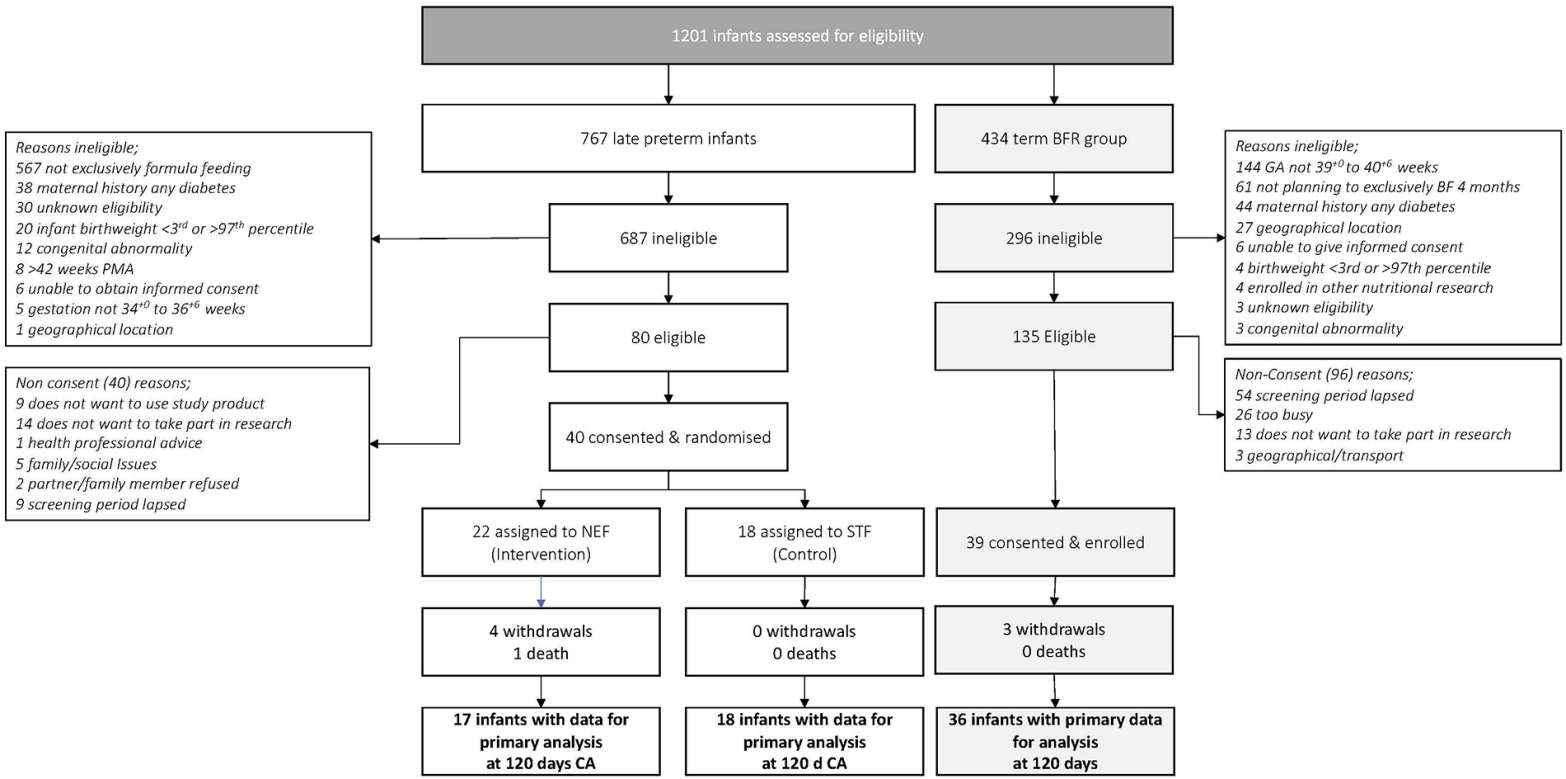
Flow of late preterm infants and term breastfeeding infants throughout the study. BFR, breastfeeding reference group; CA, corrected age; GA, gestational age; NEF, nutrient enriched formula group; STF, Standard Term Formula group.

Study recruitment began on 20th February 2018 and was terminated prematurely due to recruitment issues on the 29th August 2019. A total of 767 late preterm infants were screened with only 10.5% infants eligible which was not sustainable. The most common reason for screening failure was ‘type of feeding’ with 82.5% of infants receiving some breastmilk up to 42 weeks PMA. Of the 80 eligible infants, 40 were randomized (including 5 twin pairs), 22 to NEF and 18 to STF. 434 term breastfed infants were screened for the observational reference group and 39 were enrolled, Figure 1. Maternal characteristics were similar between randomized groups except for smoking during pregnancy STF *n* = 10, 56%; NEF *n* = 4, 18%). 77% of late preterm infants were born >35 weeks' gestation, 55% were male and mean birthweight was 3,195 grams, Table 1.

**Table 1:** Baseline maternal and infant characteristics.

	Late preterm infant group		BFR (*n* = 39)
	NEF (*n* = 22)	STF (*n* = 18)	
Hospital of birth, *n* (%)			
Other Hospital	0 (0.0)	1 (5.6)	1 (2.6)
Flinders Medical Centre	5 (22.7)	2 (11.1)	0 (0.0)
Lyell McEwin Hospital	5 (22.7)	5 (27.8)	0 (0.0)
Women's and Children's Hospital	12 (54.5)	10 (55.6)	38 (97.4)
Race^a^–European-descent Australian, *n* (%)	18 (81.8)	15 (83.3)	29 (74.4)
Maternal weight: mean (SD)	78.9 (21.4)	81.2 (16.3)	67.2 (7.6)
Maternal BMI: mean (SD)	26.2 (4.4)	30.1 (6.6)	24.9 (2.5)
Mother smoked during pregnancy, *n* (%)	4 (18.2)	10 (55.6)	2 (5.1)
Completion of secondary schooling, *n* (%)	9 (40.9)	9 (50.0)	33 (84.6)
Completion of further study, *n* (%)	13 (59.1)	12 (66.7)	37 (94.9)
Primiparous, *n* (%)	7 (31.8)	5 (27.8)	21 (53.8)
Caesarian section delivery	11 (50.0)	10 (55.6)	7 (17.9)
Infant sex male, *n* (%)	11 (50.0)	11 (61.1)	17 (43.6)
Infant from a twin birth^b^, *n* (%)	8 (36.4)	4 (22.2)	
GA late preterm infants *n* (%)			
<35 weeks	6 (27.3)	3 (16.7)	N/A
>35 weeks	16 (72.7)	15 (83.3)	N/A
PMA at enrolment (week): median (IQR)	40.0 (37.9–41.5)	40.1 (37.9–41.7)	41.4 (41.1–41.9)
Infant birth weight (gm): mean (SD)	3,203.4 (803.5)	3,187.4 (745.9)	3,602.9 (425.2)
Infant birth length (cm): mean (SD)	48.6 (2.4)	49.3 (3.5)	51.6 (2.1)
Infant birth HC (cm): mean (SD)	34.6 (2.2)	34.3 (1.9)	35.8 (1.2)
% fat: Mean (SD)	14.8 (7.3)	14.0 (6.7)	12.8 (4.0)
% fat free mass: mean (SD)	85.2 (7.3)	86.0 (6.7)	87.2 (4.0)
Fat mass: mean (SD)	0.5 (0.4)	0.5 (0.3)	0.5 (0.2)
Body mass: mean (SD)	3.2 (0.8)	3.2 (0.8)	3.6 (0.4)
Body volume: mean (SD)	3.1 (0.8)	3.1 (0.8)	3.5 (0.4)
Body density: mean (SD)	1.0 (0.0)	1.0 (0.0)	1.0 (0.0)
Fat free mass density: mean (SD)	1.1 (0.0)	1.1 (0.0)	1.1 (0.0)

BFR, breastfeeding reference group; HC, head circumference; NEF, nutrient enriched formula group; PMA, post-menstrual age; STF, standard term formula group.

^a^ Category was: European-descent Australian, European, Middle Eastern or Arabic.

^b^ NEF group includes four twin pairs, STF group Includes one twin pair and two infants from a twin birth where only one twin participated.

### 3.1. Outcomes—randomized late preterm infants

Mean (SD) infant weights at 120 days' CA in the NEF group vs. the STF group were 6,754.3 gm (744.2) and 6,645.0 gm (1,043.5), respectively. In our intention-to-treat analysis, infants in the NEF group had a mean (SD) weight gain of 29.8 (4.5) g/day compared with 28.0 (5.3) g/day for infants fed routine formula, a difference of 1.77 g/day (95% CI, −1.63 to 5.18, *P* = 0.31), Table 2. Similar results were obtained based on the per-protocol analysis, where the difference was 1.42 (95% CI, −2.08 to 4.92), *P* = 0.43. The secondary outcomes of rate of infant body length gain and head circumference gain from enrolment to 120 days' CA were similar between the randomized groups. Mean (SD) length at 120 day's CA was 62.6 cm (1.9) for NEF vs. 62.4 cm (2.8) for STF and the mean difference in rate of length gain between the NEF and STF group was 0.004 mm/day (95% CI, −0.003 to 0.0012, *P* = 0.26), Table 2. At 30 days' CA, there was a small increase in rate of length gain at in the NEF group [difference 0.014 mm/day (95% CI, 0.002–0.026), *P* = 0.02] that was not maintained at later time points, Table 2. Head circumference at 120 days' CA was comparable between the NEF and STF group measuring 41.5 cm (SD1.4) vs. 41.8 cm (SD1.1), respectively. Mean difference in head circumference gain was 0.000 mm (−0.001 to 0.001, *P* = 0.78), Table 2. Volume of study formula consumed, and number of bowel movements were comparable between the two randomized study groups at each visit, Table 3. Additional measures of tolerance differed at the 30-day visit, 7/18 infants in the NEF group were reported to have “more wind than normal” in the last 24 h compared with 0/17 infants in the STF group, Table 4. However, event numbers were small, and this difference did not persist beyond 30 days.

**Table 2:** Growth outcomes of randomized late-preterm infants from enrolment to 120 days CA.

Outcome	Days CA	NEF, Mean (SD) (*n* = 22)	STF, Mean (SD) (*n* = 18)	Mean difference^a^ (95% CI)	*P*-value
Rate of weight gain (g/day since enrolment)	30	36.8 (7.4)	36.0 (7.1)	0.77 (−4.13, 5.67)	0.76
	60	34.2 (5.0)	33.2 (5.1)	1.01 (−2.38, 4.40)	0.56
	90	31.5 (4.6)	30.3 (5.1)	1.18 (−2.16, 4.51)	0.49
	120	29.8 (4.5)	28.0 (5.3)	1.77 (−1.63, 5.18)	0.31
Rate of length gain (mm/day since enrolment)	30	0.14 (0.01)	0.12 (0.02)	0.014 (0.002, 0.026)	0.02
	60	0.12 (0.01)	0.12 (0.02)	0.004 (−0.005, 0.014)	0.38
	90	0.12 (0.01)	0.12 (0.02)	0.004 (−0.007, 0.014)	0.48
	120	0.11 (0.01)	0.11 (0.01)	0.004 (−0.003, 0.012)	0.26
Rate of HC gain (mm/day since enrolment)	30	0.09 (0.00)	0.09 (0.00)	0.000 (−0.000, 0.001)	0.27
	60	0.07 (0.00)	0.07 (0.00)	−0.001 (−0.003, 0.002)	0.72
	90	0.07 (0.00)	0.07 (0.00)	0.000 (−0.001, 0.001)	0.51
	120	0.06 (0.00)	0.06 (0.00)	0.000 (−0.001, 0.001)	0.78

CA, corrected age, HC, head circumference; NEF, nutrient enriched formula; STF, standard term formula**.**

^a^ Mean difference between NEF and STF (i.e. NEF—STF), adjusted for infant sex.

**Table 3:** Formula intake and bowel movements.

Outcome	Days CA	NEF, Mean (SD, *n*)	STF, Mean (SD, *n*)	Mean difference^a^ (95% CI)	*P*-value
Volume of study formula consumed (mls)^b^	30	706.9 (407.4, *n* = 17)	767.5 (342.7, *n* = 17)	−60.63 (−342.89, 221.64)	0.67
	60	774.6 (357.1, *n* = 13)	619.4 (422.5, *n* = 17)	155.24 (−119.06, 429.54)	0.27
	90	905.4 (262.9, *n* = 14)	843.0 (385.7, *n* = 16)	62.42 (−174.29, 299.13)	0.61
	120	964.6 (166.2^b^, *n* = 14)	840.7 (413.1^c^, *n* = 15)	123.92 (−94.81, 342.65)	0.27
Number of bowel movements	30	2.1 (1.4, *n* = 16)	1.6 (0.6, *n* = 16)	0.50 (−0.22, 1.22)	0.17
	60	2.0 (1.5, *n* = 10)	1.6 (0.8, *n* = 13)	0.38 (−0.59, 1.36)	0.44
	90	1.7 (0.9, *n* = 13)	1.6 (0.5, *n* = 14)	0.12 (−0.41, 0.65)	0.65
	120	1.8 (0.8, *n* = 12)	1.3 (0.5, *n* = 14)	0.46 (−0.00, 0.93)	0.05

CA, corrected age, HC, head circumference; NEF, nutrient enriched formula; STF, standard term formula.

^a^ Mean difference between NEF and STF (i.e. NEF-STF), adjusted for infant sex.

^b^ Only includes infants who were still consuming formula at the corresponding timepoint.

^c^ Large differences in SD between STF and NEF driven by 2 participants who consumed 0 mls in the last 24 h in the STF group but still indicated they were taking study formula.

**Table 4:** Formula tolerance.

Outcome	Day	Labels	NEF, *N* (%) (*n* = 22)	STF, *N* (%) (*n* = 18)	*P*-value*
Stool consistency	30				0.84
		Missing^a^	6 (27.3)	2 (11.1)	
		Type 2: Formed, definite shape, not dry	1 (4.5)	2 (11.1)	
		Type 3: Soft—no definite shape, pasty	11 (50.0)	12 (66.7)	
		Type 4: Unformed or seedy	3 (13.6)	1 (5.6)	
		Type 5: Watery, no shape, mainly water	1 (4.5)	1 (5.6)	
	60				1
		Missing^a^	12 (54.5)	5 (27.8)	
		Type 2: Formed, definite shape, not dry	0 (0.0)	1 (5.6)	
		Type 3: Soft—no definite shape, pasty	8 (36.4)	9 (50.0)	
		Type 4: Unformed or seedy	2 (9.1)	3 (16.7)	
		Type 5: Watery, no shape, mainly water	0 (0.0)	0 (0.0)	
	90				0.53
		Missing^a^	9 (40.9)	4 (22.2)	
		Type 2: Formed, definite shape, not dry	2 (9.1)	1 (5.6)	
		Type 3: Soft—no definite shape, pasty	9 (40.9)	12 (66.7)	
		Type 4: Unformed or seedy	2 (9.1)	1 (5.6)	
		Type 5: Watery, no shape, mainly water	0 (0.0)	0 (0.0)	
	120				0.41
		Missing^a^	10 (45.5)	4 (22.2)	
		Type 2: Formed, definite shape, not dry	2 (9.1)	1 (5.6)	
		Type 3: Soft—no definite shape, pasty	6 (27.3)	9 (50.0)	
		Type 4: Unformed or seedy	2 (9.1)	4 (22.2)	
		Type 5: Watery, no shape, mainly water	2 (9.1)	0 (0.0)	
How settled the infant was	30				0.37
		Missing^a^	4 (18.2)	1 (5.6)	
		Less unsettled than normal	1 (4.5)	0 (0.0)	
		About as unsettled as normal	13 (59.1)	10 (55.6)	
		More unsettled than normal	4 (18.2)	7 (38.9)	
	60				0.84
		Missing^a^	7 (31.8)	1 (5.6)	
		Less unsettled than normal	1 (4.5)	1 (5.6)	
		About as unsettled as normal	11 (50.0)	11 (61.1)	
		More unsettled than normal	3 (13.6)	5 (27.8)	
	90				0.33
		Missing^a^	7 (31.8)	2 (11.1)	
		Less unsettled than normal	1 (4.5)	0 (0.0)	
		About as unsettled as normal	13 (59.1)	12 (66.7)	
		More unsettled than normal	1 (4.5)	4 (22.2)	
	120				0.69
		Missing^a^	8 (36.4)	2 (11.1)	
		Less unsettled than normal	1 (4.5)	1 (5.6)	
		About as unsettled as normal	11 (50.0)	10 (55.6)	
		More unsettled than normal	2 (9.1)	5 (27.8)	
Wind in the last 24 h	30				0.008
		Missing^a^	4 (18.2)	1 (5.6)	
		Less wind than normal	0 (0.0)	1 (5.6)	
		About the same amount of wind as normal	11 (50.0)	16 (88.9)	
		More wind than normal	7 (31.8)	0 (0.0)	
	60				0.84
		Missing^a^	7 (31.8)	1 (5.6)	
		Less wind than normal	2 (9.1)	2 (11.1)	
		About the same amount of wind as normal	12 (54.5)	12 (66.7)	
		More wind than normal	1 (4.5)	3 (16.7)	
	90				0.69
		Missing^a^	7 (31.8)	2 (11.1)	
		Less wind than normal	1 (4.5)	3 (16.7)	
		About the same amount of wind as normal	12 (54.5)	12 (66.7)	
		More wind than normal	2 (9.1)	1 (5.6)	
	120				0.03
		Missing^a^	8 (36.4)	2 (11.1)	
		Less wind than normal	1 (4.5)	6 (33.3)	
		About the same amount of wind as normal	13 (59.1)	8 (44.4)	
		More wind than normal	0 (0.0)	2 (11.1)	

NEF, nutrient enriched formula; STF, standard term formula.

^a^ Missing values excluded from analysis.

* *P*-values from Chi-square (or Fisher's exact test where cell counts are low) with no accounting for clustering due to twins.

### 3.2. Outcomes—all infants

There were no differences between randomized groups on weight, length and head circumference *z*-scores at 365 days of age. When compared with the BFR group, infant weight and length *z*-scores were comparable, however, head circumference was smaller in infants randomized to the STF group compared to the BFR group (adjusted mean difference (AMD) −1.09, 95% CI, −1.79 to −0.40, *P* = 0.002, Table 5. Mean percentages of fat in late preterm infants at 120 days' CA were similar between the NEF (24.6%) and STF groups (24.4%), AMD 1.41, 95% CI, −3.40 to 6.22, *P* = 0.56, Table 6. Differences between randomized groups and the BFR group were evident in the NEF group only, which showed increased fat free mass (AMD 0.46 Kg, 95% CI, 0.11–0.81, *P* = 0.01), body mass (AMD 0.98 Kg, 95% CI, 0.18, 1.78, *P* = 0.02) and body volume (AMD 1.01 L, 95% CI, 0.18–1.84, *P* = 0.02) compared to breastfeeding infants, Table 6. There were no differences in SCORAD objective scores between the groups at any timepoint, Table 7. Adverse events were comparable across all groups, occurring in 96.2% of infants by 365 days (CA for preterm infants), Table 8. Further analysis of pre-defined subgroups of AEs was undertaken including diarrhea, infectious illness (any bacterial or viral illness) and otitis media, as well as the post-hoc outcome of respiratory illness. Comparisons, adjusted for index of relative socio-economic disadvantage quintiles showed a significant reduction in “infectious illness” at 120 days in infants fed NEF compared to STF [relative risk (RR) 0.37, 95% CI, 0.16, 0.85, *P* = 0.02] and NEF compared to term BFR group (RR 0.37, 95% CI, 0.17, 0.81, *P* = 0.01), Table 8. Serious adverse events occurred in one infant in the NEF group, five in the STF group and three in the BFR reference group, Table 8. All serious adverse events were classified as unrelated to the study product or study protocol.

**Table 5:** Growth outcomes of randomized late preterm infants and term breastfeeding reference group.

Outcome	Timepoint	Comparison	Unadjusted Mean difference (95% CI)	*P*-value	Adjusted^a^ Mean difference (95% CI)	*P*-value
Weight *z*-score	30	STF—BFR	−1.69 (−2.22, −1.16)	<0.0001	−1.56 (−2.00, −1.12)	<0.0001
		NEF—BFR	−1.55 (−2.09, −1.01)	<0.0001	−1.07 (−1.53, −0.62)	<0.0001
		NEF—STF	0.14 (−0.56, 0.83)	0.70	0.49 (−0.07, 1.04)	0.09
		Overall		<0.0001		<0.0001
	60	STF—BFR	−1.25 (−1.77, −0.72)	<0.0001	−1.10 (−1.56, −0.64)	<0.0001
		NEF—BFR	−1.05 (−1.63, −0.46)	0.0005	−0.44 (−1.01, 0.13)	0.13
		NEF—STF	0.20 (−0.53, 0.93)	0.59	0.66 (0.01, 1.31)	0.05
		Overall		<0.0001		<0.0001
	90	STF—BFR	−0.87 (−1.48, −0.25)	0.006	−0.60 (−1.23, 0.03)	0.06
		NEF—BFR	−0.69 (−1.36, −0.03)	0.04	0.04 (−0.69, 0.76)	0.92
		NEF—STF	0.17 (−0.67, 1.01)	0.69	0.64 (−0.19, 1.47)	0.13
		Overall		0.005		0.14
	120	STF—BFR	−0.62 (−1.36, 0.12)	0.10	−0.30 (−1.07, 0.47)	0.44
		NEF—BFR	−0.39 (−1.05, 0.28)	0.26	0.32 (−0.52, 1.15)	0.46
		NEF—STF	0.24 (−0.68, 1.15)	0.61	0.62 (−0.46, 1.69)	0.26
		Overall		0.17		0.53
	180	STF—BFR	−0.20 (−0.79, 0.40)	0.52	0.05 (−0.61, 0.70)	0.89
		NEF—BFR	−0.19 (−0.95, 0.57)	0.63	0.51 (−0.67, 1.68)	0.40
		NEF—STF	0.01 (−0.88, 0.90)	0.99	0.46 (−0.84, 1.76)	0.49
		Overall		0.75		0.70
	365	STF—BFR	0.02 (−0.45, 0.48)	0.95	0.42 (−0.19, 1.04)	0.17
		NEF—BFR	0.08 (−0.71, 0.88)	0.84	1.16 (−0.19, 2.51)	0.09
		NEF—STF	0.07 (−0.77, 0.91)	0.87	0.73 (−0.51, 1.97)	0.25
		Overall		0.98		0.18
Length *z*-score	30	STF—BFR	−2.34 (−2.86, −1.81)	<0.0001	−2.14 (−2.53, −1.76)	<0.0001
		NEF—BFR	−2.14 (−2.63, −1.65)	<0.0001	−1.60 (−2.14, −1.05)	<0.0001
		NEF—STF	0.19 (−0.42, 0.80)	0.53	0.55 (−0.03, 1.12)	0.06
		Overall		<0.0001		<0.0001
	60	STF—BFR	−1.83 (−2.38, −1.27)	<0.0001	−1.49 (−1.94, −1.04)	<0.0001
		NEF—BFR	−1.89 (−2.41, −1.37)	<0.0001	−1.33 (−2.03, −0.63)	0.0002
		NEF—STF	−0.06 (−0.73, 0.60)	0.85	0.16 (−0.53, 0.85)	0.65
		Overall		<0.0001		<0.0001
	90	STF—BFR	−1.44 (−2.19, −0.70)	0.0001	−1.18 (−1.76, −0.60)	<0.0001
		NEF—BFR	−1.32 (−1.98, −0.66)	<0.0001	−0.83 (−1.82, 0.16)	0.10
		NEF—STF	0.12 (−0.77, 1.02)	0.78	0.36 (−0.68, 1.40)	0.50
		Overall		<0.0001		0.0002
	120	STF—BFR	−1.24 (−1.91, −0.57)	0.0003	−0.78 (−1.45, −0.11)	0.02
		NEF—BFR	−1.06 (−1.68, −0.45)	0.0007	−0.17 (−1.03, 0.69)	0.70
		NEF—STF	0.18 (−0.62, 0.98)	0.66	0.61 (−0.38, 1.60)	0.22
		Overall		<0.0001		0.07
	180	STF—BFR	−0.60 (−1.29, 0.08)	0.08	−0.17 (−0.78, 0.43)	0.58
		NEF—BFR	−0.78 (−1.41, −0.15)	0.02	0.06 (−0.79, 0.92)	0.88
		NEF—STF	−0.18 (−0.99, 0.64)	0.67	0.24 (−0.69, 1.16)	0.62
		Overall		0.03		0.82
	365	STF—BFR	−0.49 (−1.54, 0.56)	0.36	0.30 (−1.49, 2.09)	0.74
		NEF—BFR	−0.50 (−1.25, 0.26)	0.20	0.93 (−0.16, 2.02)	0.10
		NEF—STF	−0.01 (−1.19, 1.18)	0.99	0.62 (−1.40, 2.65)	0.55
		Overall		0.34		0.24
Head circumference *z*-score	30	STF—BFR	−1.70 (−2.24, −1.17)	<0.0001	−1.96 (−2.68, −1.24)	<0.0001
		NEF—BFR	−1.25 (−1.78, −0.73)	<0.0001	−1.06 (−1.63, −0.50)	0.0002
		NEF—STF	0.45 (−0.22, 1.12)	0.19	0.90 (0.18, 1.61)	0.01
		Overall		<0.0001		<0.0001
	60	STF—BFR	−1.19 (−1.75, −0.64)	<0.0001	−1.56 (−2.14, −0.99)	<0.0001
		NEF—BFR	−1.27 (−1.75, −0.79)	<0.0001	−1.02 (−1.70, −0.34)	0.003
		NEF—STF	−0.08 (−0.73, 0.57)	0.81	0.54 (−0.15, 1.23)	0.12
		Overall		<0.0001		<0.0001
	90	STF—BFR	−0.77 (−1.34, −0.20)	0.008	−1.27 (−1.98, −0.56)	0.0005
		NEF—BFR	−0.81 (−1.36, −0.26)	0.004	−0.87 (−1.59, −0.15)	0.02
		NEF—STF	−0.04 (−0.75, 0.68)	0.92	0.40 (−0.41, 1.21)	0.34
		Overall		0.002		0.001
	120	STF—BFR	−0.42 (−0.98, 0.14)	0.14	−0.86 (−1.54, −0.18)	0.01
		NEF—BFR	−0.59 (−1.15, −0.04)	0.04	−0.52 (−1.36, 0.32)	0.22
		NEF—STF	−0.17 (−0.87, 0.52)	0.63	0.34 (−0.52, 1.20)	0.44
		Overall		0.07		0.04
	180	STF—BFR	−0.18 (−0.73, 0.37)	0.52	−0.45 (−1.02, 0.12)	0.12
		NEF—BFR	−0.47 (−1.01, 0.07)	0.09	−0.42 (−1.15, 0.32)	0.27
		NEF—STF	−0.29 (−0.96, 0.38)	0.40	0.03 (−0.71, 0.77)	0.94
		Overall		0.23		0.26
	365	STF—BFR	−0.43 (−1.10, 0.23)	0.20	−1.09 (−1.79, −0.40)	0.002
		NEF—BFR	−0.44 (−1.19, 0.30)	0.24	−0.30 (−0.97, 0.38)	0.39
		NEF—STF	−0.01 (−0.88, 0.86)	0.98	0.80 (−0.04, 1.63)	0.06
		Overall		0.30		0.008

BFR, breastfeeding reference; NEF, nutrient enriched formula; STF, standard term formula.

^a^ Adjusted for index of relative socio-economic disadvantage quintile and maternal BMI (not infant sex as this is already incorporated in the *z*-score calculation).

**Table 6:** Body Composition of all groups at 120 days of age.

Outcome	Comparison	Unadjusted Mean difference (95% CI)	*P*-value	Adjusted^a^ Mean difference (95% CI)	*P*-value
%fat	STF—BFR	−0.72 (−3.87, 2.42)	0.65	2.18 (−1.46, 5.83)	0.24
	NEF—BFR	−0.43 (−4.37, 3.51)	0.83	3.60 (−0.28, 7.47)	0.07
	NEF—STF	0.29 (−4.32, 4.91)	0.90	1.41 (−3.40, 6.22)	0.56
	Overall		0.89		0.13
%fat free mass	STF—BFR	0.72 (−2.42, 3.87)	0.65	−2.18 (−5.83, 1.46)	0.24
	NEF—BFR	0.43 (−3.51, 4.37)	0.83	−3.60 (−7.47, 0.28)	0.07
	NEF—STF	−0.29 (−4.91, 4.32)	0.90	−1.41 (−6.22, 3.40)	0.56
	Overall		0.89		0.13
Fat mass (kg)	STF—BFR	0.05 (−0.27, 0.38)	0.74	0.29 (−0.06, 0.63)	0.10
	NEF—BFR	0.06 (−0.33, 0.44)	0.78	0.52 (0.04, 1.00)	0.03
	NEF—STF	0.00 (−0.47, 0.48)	1.00	0.23 (−0.30, 0.76)	0.39
	Overall		0.92		0.05
Fat free mass (kg)^b^	STF—BFR	0.28 (−0.13, 0.70)	0.18	0.19 (−0.17, 0.56)	0.30
	NEF—BFR	0.23 (−0.02, 0.47)	0.07	0.46 (0.11, 0.81)	0.01
	NEF—STF	−0.06 (−0.48, 0.37)	0.79	0.27 (−0.12, 0.65)	0.18
	Overall		0.13		0.04
Body mass (kg)	STF—BFR	0.34 (−0.31, 0.99)	0.31	0.48 (−0.09, 1.04)	0.10
	NEF—BFR	0.28 (−0.22, 0.78)	0.27	0.98 (0.18, 1.78)	0.02
	NEF—STF	−0.06 (−0.82, 0.70)	0.88	0.50 (−0.33, 1.33)	0.24
	Overall		0.37		0.03
Body volume (L)	STF—BFR	0.33 (−0.33, 0.98)	0.33	0.50 (−0.08, 1.08)	0.09
	NEF—BFR	0.27 (−0.25, 0.80)	0.31	1.01 (0.18, 1.84)	0.02
	NEF—STF	−0.05 (−0.84, 0.73)	0.89	0.51 (−0.35, 1.37)	0.25
	Overall		0.42		0.03
Body density (kg/L)	STF—BFR	0.00 (−0.00, 0.01)	0.64	0.00 (−0.01, 0.00)	0.25
	NEF—BFR	0.00 (−0.01, 0.01)	0.82	−0.01 (−0.01, 0.00)	0.07
	NEF—STF	−0.00 (−0.01, 0.01)	0.90	0.00 (−0.01, 0.01)	0.56
	Overall		0.89		0.14
Fat mass *z*-score^b^	STF—BFR	−0.32 (−1.09, 0.44)	0.41	0.24 (−0.54, 1.01)^c^	0.55
	NEF—BFR	−0.32 (−1.30, 0.66)	0.52	0.84 (−0.36, 2.05)^c^	0.17
	NEF—STF	0.00 (−1.17, 1.18)	1.00	0.60 (−0.70, 1.90)^c^	0.36
	Overall		0.61		0.37
Fat free mass *z*-score^b^	STF—BFR	−0.21 (−1.00, 0.58)	0.61	−0.32 (−1.17, 0.53)^c^	0.46
	NEF—BFR	−0.32 (−0.99, 0.36)	0.36	0.35 (−0.61, 1.32)^c^	0.47
	NEF—STF	−0.11 (−1.03, 0.82)	0.82	0.68 (−0.38, 1.73)^c^	0.21
	Overall		0.62		0.45
%fat *z*-score^b^	STF—BFR	−0.19 (−0.84, 0.45)	0.56	0.36 (−0.43, 1.15)^c^	0.37
	NEF—BFR	−0.10 (−0.79, 0.60)	0.79	0.70 (−0.05, 1.45)^c^	0.07
	NEF—STF	0.10 (−0.77, 0.96)	0.82	0.34 (−0.63, 1.30)^c^	0.49
	Overall		0.83		0.16

BFR, breastfeeding reference group; NEF, nutrient enriched formula group; STF, standard term formula group.

^a^ Adjusted for index of relative socio-economic disadvantage quintile, infant sex and maternal BMI unless otherwise specified.

^b^ Post-hoc outcome analyzed after the pre-specified analysis was completed.

^c^ Adjusted for index of relative socio-economic disadvantage quintile and maternal BMI (not infant sex as this is already incorporated in the *z*-score calculation).

**Table 7:** SCORAD^a^ assessment all groups.

Outcome	Timepoint	Comparison	Unadjusted Mean difference (95% CI)	*P*-value	Adjusted^b^ Mean difference (95% CI)	*P*-value
Objective score	120	STF—BFR	−0.47 (−2.07, 1.14)	0.57	0.18 (−1.34, 1.71)	0.81
		NEF—BFR	3.87 (−2.55, 10.30)	0.24	5.43 (−1.79, 12.65)	0.14
		NEF—STF	4.34 (−1.94, 10.62)	0.18	5.25 (−1.63, 12.13)	0.13
		Overall		0.35		0.33
	180	STF—BFR	−0.28 (−0.82, 0.26)	0.31	0.09 (−0.57, 0.75)	0.80
		NEF—BFR	1.72 (−2.11, 5.55)	0.38	2.26 (−1.96, 6.48)	0.29
		NEF—STF	2.00 (−1.79, 5.79)	0.30	2.17 (−1.82, 6.17)	0.29
		Overall		0.35		0.57

BFR, breastfeeding reference group; HC, head circumference; NEF, nutrient enriched formula group; STF, standard term formula group; SCORAD, SCORing Atopic Dermatitis assessment.

^a^ Results at days 30, 60, 90 and 365 days not reported due to insufficient number of participants with SCORAD assessment.

^b^ Adjusted for index of relative socio-economic disadvantage quintile.

**Table 8:** Adverse events.

Outcome	Timepoint	Comparison	Unadjusted RR (95% CI)	*P*-value	Adjusted^a^ RR (95% CI)	*P*-value
Adverse Events all groups^b^						
Adverse Events	120	STF vs BFR	1.03 (0.98, 1.08)	0.32	1.05 (0.97, 1.14)	0.24
		NEF vs BFR	0.93 (0.77, 1.13)	0.48	0.95 (0.81, 1.11)	0.54
		NEF vs STF	0.91 (0.76, 1.09)	0.31	0.91 (0.77, 1.07)	0.25
		Overall		0.36		0.36
	365	STF vs BFR	1.03 (0.98, 1.08)	0.32	1.05 (0.97, 1.14)	0.24
		NEF vs BFR	0.93 (0.77, 1.13)	0.48	0.95 (0.81, 1.11)	0.54
		NEF vs STF	0.91 (0.76, 1.09)	0.31	0.91 (0.77, 1.07)	0.25
		Overall		0.36		0.36
Serious Adverse Events	120	STF vs BFR	5.42 (1.18, 24.81)	0.03	4.67 (1.05, 20.77)	0.04
		NEF vs BFR	0.89 (0.08, 9.35)	0.92	0.65 (0.06, 6.61)	0.72
		NEF vs STF	0.16 (0.02, 1.28)	0.08	0.14 (0.02, 1.04)	0.06
		Overall		0.04		0.03
	365	STF vs BFR	3.61 (0.99, 13.17)	0.05	2.85 (0.75, 10.90)	0.13
		NEF vs BFR	0.59 (0.06, 5.42)	0.64	0.44 (0.05, 3.80)	0.45
		NEF vs STF	0.16 (0.02, 1.28)	0.08	0.15 (0.02, 1.16)	0.07
		Overall		0.06		0.10
Pre-defined subgroup of Adverse Events						
Infectious illness^c^	120	STF vs BFR	1.13 (0.74, 1.73)	0.57	0.99 (0.66, 1.49)	0.95
		NEF vs BFR	0.46 (0.20, 1.06)	0.07	0.37 (0.17, 0.81)	0.01
		NEF vs STF	0.41 (0.17, 0.97)	0.04	0.37 (0.16, 0.85)	0.02
		Overall		0.13		0.04
	365	STF vs BFR	1.13 (0.77, 1.64)	0.53	1.02 (0.71, 1.47)	0.91
		NEF vs BFR	0.50 (0.24, 1.04)	0.06	0.44 (0.21, 0.88)	0.02
		NEF vs STF	0.44 (0.21, 0.94)	0.03	0.43 (0.20, 0.89)	0.02
		Overall		0.11		0.06
Other illness	120	STF vs BFR	1.39 (0.73, 2.65)	0.31	1.65 (0.87, 3.15)	0.13
		NEF vs BFR	1.39 (0.73, 2.65)	0.31	1.63 (0.83, 3.20)	0.15
		NEF vs STF	1.00 (0.50, 1.99)	1.00	0.99 (0.50, 1.94)	0.97
		Overall		0.49		0.23
	365	STF vs BFR	1.22 (0.66, 2.26)	0.53	1.44 (0.78, 2.64)	0.24
		NEF vs BFR	1.22 (0.66, 2.26)	0.53	1.39 (0.72, 2.68)	0.33
		NEF vs STF	1.00 (0.50, 1.99)	1.00	0.96 (0.49, 1.89)	0.91
		Overall		0.75		0.44
Respiratory illness^d^	120	STF vs BFR	1.44 (0.80, 2.60)	0.22	1.32 (0.71, 2.44)	0.38
		NEF vs BFR	0.59 (0.22, 1.59)	0.30	0.51 (0.19, 1.37)	0.18
		NEF vs STF	0.41 (0.15, 1.12)	0.08	0.39 (0.15, 1.04)	0.06
		Overall		0.17		0.16
	365	STF vs BFR	1.32 (0.79, 2.21)	0.28	1.23 (0.72, 2.09)	0.44
		NEF vs BFR	0.59 (0.25, 1.40)	0.23	0.55 (0.24, 1.29)	0.17
		NEF vs STF	0.45 (0.19, 1.07)	0.07	0.45 (0.19, 1.06)	0.07
		Overall		0.17		0.19

BFR, breastfeeding reference group; NEF, nutrient enriched formula group; STF, standard term formula group.

^a^ Adjusted for index of relative socio-economic disadvantage quintile.

^b^ Adverse Events can be classified into multiple subtypes; No analysis performed for subtypes of Adverse Events or Serious Adverse Events due to small numbers.

^c^ Infectious illness includes all illness with bacterial or viral origin.

^d^ Post-hoc outcome analyzed after the pre-specified analysis was completed. Respiratory illness includes acute upper respiratory infections, Influenza and pneumonia, and other acute lower respiratory infections.

Vitamin D status, as measured by 250HD3-PTAD, was higher in the NEF group 78.7 nmol/L compared to both the STF 68.9 nmol/L and reference group 43.1 nmol/L, *P* < 0.0001, (data not shown). There were no significant differences between all groups in butyrate, the AMD between the NEF and STF groups was −0.54 µmol/L (95% CI, −3.03 to 1.96), *P* = 0.67 (data not shown). There were no significant differences between the NEF and STF groups in fecal microbiota measures of total bacterial load, microbiota structure (richness and diversity) and composition at 60 days and 120 days of age (data not shown). In comparison to the BFR group, microbial richness was significantly higher in both the NEF and STF groups and microbiota composition significantly differed to both groups at 60 and 120 days of age, Table 9. Dynamic changes in the relative abundances of taxa contributing to these differences and microbiota compositional differences are reported in the Supplementary Figure 1. Sequencing data is publicly available from Sequence Read Archive (SRA) NCBI under the Bioproject accession number PRJNA864095.

**Table 9:** Fecal microbiome analysis of all groups at enrolment, 60 days and 120 days of age.

Outcome	Timepoint	Group	Median (95% CI)	Group comparisons	
				Comparison	Adjusted^a^ *P*-value
Total bacterial load (16S copies/g feces)	Enrolment	STF	5.3 × 10^5^ (2.7 × 10^5^, 2.4 × 10^6^)	STF-BFR	0.147
		NEF	1.0 × 10^6^ (3.9 × 10^5^, 1.6 × 10^6^)	NEF-BFR	0.103
		BFR	2.7 × 10^5^ (1.3 × 10^5^, 4.7 × 10^5^)	NEF-STF	0.776
	60	STF	7.4 × 10^5^ (1.1 × 10^5^, 2.6 × 10^6^)	STF-BFR	0.820
		NEF	1.9 × 10^6^ (4.0 × 10^4^, 1.3 × 10^6^)	NEF-BFR	0.289
		BFR	4.1 × 10^5^ (1.9 × 10^5^, 1.4 × 10^6^)	NEF-STF	0.289
	120	STF	4.8 × 10^5^ (8.2 × 10^4^, 5.2 × 10^6^)	STF-BFR	0.983
		NEF	7.6 × 10^5^ (6.9 × 10^4^, 5.0 × 10^6^)	NEF-BFR	0.983
		BFR	5.0 × 10^5^ (2.4 × 10^5^, 1.4 × 10^6^)	NEF-STF	0.983
Microbial richness (Observed species)	Enrolment	STF	49 (40, 57)	STF-BFR	0.015
		NEF	43 (36, 56)	NEF-BFR	0.024
		BFR	38 (33, 41)	NEF-STF	0.619
	60	STF	67 (55, 72)	STF-BFR	<0.001
		NEF	65 (62, 82)	NEF-BFR	<0.001
		BFR	37 (32, 44)	NEF-STF	0.391
	120	STF	69 (54, 70)	STF-BFR	<0.001
		NEF	72 (63, 90)	NEF-BFR	<0.001
		BFR	48 (36, 54)	NEF-STF	0.091
Microbial diversity (Faith's phylogenetic diversity)	Enrolment	STF	8.9 (5.2, 11.8)	STF-BFR	0.864
		NEF	7.3 (4.3, 10.8)	NEF-BFR	0.864
		BFR	8.1 (5.5, 10.9)	NEF-STF	0.864
	60	STF	8.7 (7.6, 10.2)	STF-BFR	0.428
		NEF	7.3 (6.0, 10.7)	NEF-BFR	0.524
		BFR	7.3 (5.7, 9.3)	NEF-STF	0.524
	120	STF	8.1 (6.7, 9.2)	STF-BFR	0.368
		NEF	9.0 (6.2, 12.7)	NEF-BFR	0.106
		BFR	6.9 (4.3, 9.8)	NEF-STF	0.368

BFR, breastfeeding reference; NEF, nutrient enriched formula; STF, standard term formula.

^a^ Adjusted based on the false discovery rate method for multiple comparisons.

## 4. Discussion

We aimed to investigate the effect of feeding late preterm infants a nutrient enriched formula on rate of weight gain in comparison to standard term formula. We saw no evidence to suggest an effect on rate of body weight gain from enrolment to 120 days' CA in the NEF group compared to STF group.

This study was designed to detect a difference of 3 g/day between the NEF and STF groups and our estimated difference of 1.77 g/day is well below this. However, the significant reduction in sample size because of recruitment challenges increases our chance of a null finding. We successfully screened 767 late preterm infants in an 18-month period but only 10.5% of infants were eligible to participate and test the efficacy and safety of our nutrient enriched formula, even though these are the infants who are predominantly fed such products. We found high rates of breastfeeding (partial or exclusive) during initial hospitalization and in the immediate discharge period. A cohort study, conducted during a similar time period to this trial, showed that 15% of late preterm infants were exclusively formula feeding at discharge, increasing to 27% at 6 weeks of age (25). Future protocols investigating nutrient enriched formulas could consider extending the study eligibility period beyond the 42-week post-menstrual age cut off adhered to in this study.

Our findings are consistent with the most recent Cochrane review comparing nutrient enriched formula vs. standard term formula, for preterm infants following hospital discharge (12). Young et al. included 15 trials and concluded that there was no strong or consistent evidence that unrestricted feeding with nutrient-enriched formula affects growth and development up to about 18 months of age (12). However, there are several limitations of meta-analyses due to the marked heterogeneity between studies with regard to population characteristics, inclusion criteria, formula composition, and initiation and duration of the study feeding (11). Furthermore, trials included the Cochrane Review and a more recent systematic review (26) have focused on the very preterm or very low birthweight infant, hence there is a gap in the evidence for AGA late preterm infants that our trial intended to address. There has been suggestion that null findings in nutrient enriched formula studies may be a result of infants regulating their formula consumption in relation to the energy or nutrient density of the formula (27–30). This was not the case in our study, where volume of formula intake from enrolment to 120 days’ CA did not appear to be affected by formula composition and was comparable between the NEF and STF groups.

There was some evidence of a difference between the randomized groups or in comparison with the breastfed reference group in some of our secondary outcomes. These results should be interpreted with caution based on the small sample size, large number of statistical tests performed, lack of adjustment for multiple comparisons and lack of control for all potential confounders in comparisons with the reference group (due to the small sample size). Infants fed NEF showed a small increase in rate of length gain at 30 days' CA compared with the STF group, but this difference was not maintained at subsequent study visits. In studies of very low birth weight infants, early differences in gains suggest a particularly important effect of nutrient-enriched feedings during the early post-discharge period (30), but could be a chance finding in our study. When compared to the BFR group, head circumference *Z*-scores at 365 days CA in the NEF group were comparable. In contrast, head circumference *z*-scores of infants in the STF group were lower compared to the BFR group. There is suggestion that the first year of life provides an important opportunity for human somatic and brain growth to compensate for earlier deprivation (31). Often the most significant effects of nutrient enriched formulas are seen in infants with the lowest birthweights (30) and there may be benefits for late preterm infants with low birthweight (not appropriately grown for gestational age), although these infants were excluded from participating in the current study. Concern has been expressed that “recovery” or “catch-up” growth as a result of feeding nutrient enriched formula during infancy may be associated with the subsequent development of insulin resistance and central adiposity in preterm infants (32, 33). Our data do not support this as we saw no evidence to suggest any difference in body composition between the NEF and STF groups. Rather, differences in body composition were evident in the NEF group compared to the BFR group at 120 days with increased fat free mass, body mass and body volume in the NEF compared to the BFR group.

Formula consumption (ml/day) was not affected by assignment to either NEF or STF, indicating a good acceptance and palatability of NEF relative to STF. Stool consistency was comparable between groups although infants fed NEF had slightly more wind in the first 120 days of the study feeding, which could be a chance finding. Fecal microbiota of the NEF and STF groups were comparable at all timepoints, although significant microbiota differences were observed when compared to the reference BFR group. The type of infant milk feed can influence gut microbiota composition, either by providing substrates for bacterial growth and function or by acting as a source of bacterial populations (such as bacteria in the surrounding skin during breast-feeding or in the formula milk suspension) (34). Nevertheless, the impact of formula-feeding (NEF or STF) on the gut microbiota compared to the breastfed infants in the reference group were consistent with previous studies. In particular, the lack of difference in *Bifidobacterium* abundance between formula- and breastfed infants (35, 36), as well as a lower number of observed species and increases in the relative abundance of the skin bacteria *Staphylococcus* (37) and *Cutibacterium* in breastfed infants, align with previously reported effects.

Nutrient enriched formulas designed for preterm infants following discharge from hospital are variably enriched with minerals, vitamins and trace elements compared with standard term formula. In addition to increased vitamin D, our nutrient enriched formula composition also had added bovine MFGM. Formula fed infants are of special interest with respect to added dietary MFGM since they have a lower intake of MFGM components compared to breastfed infants (38). Studies investigating the effect of dietary bovine MFGM use infants and children have shown some promise against infections, however interventions and outcome measures are heterogeneous (38). In the present study there was a significant reduction in “any infectious illness” in infants fed NEF compared to both the STF (63% reduction) and BFR (63% reduction) groups through to 120 days' CA. Although adverse events were adjusted for Socio-Economic Indexes For Areas (SEIFA), comparisons between formula feeding late-preterm infants and term breastfeeding infants have limitations because of residual confounding due to confounders we could not adjust for (due to small sample size) or did not measure. Our findings of a reduction in infectious illness in the NEF group are consistent with some other studies (39–41), however, further adequately powered, high-quality trials are needed before firm conclusions can be drawn on benefits of added bovine MFGM for this population (38). Increased vitamin D status in infants fed NEF may also have been a contributing factor in the reduction in infectious illness. Mean vitamin-D levels in the NEF group were 1.8 times higher than the reference group (78.7 nmol/L vs. 43.1 nmol/L) and only marginally higher than the STF group (68.9 nmol/L). Epidemiological evidence has suggested that low vitamin-D status is associated with an increased risk of respiratory tract infections in children (42). Although RCT evidence is limited, one recent study found a six-fold increased risk of infant respiratory syncytial virus associated lower respiratory tract infections in infants with lower vitamin D status at birth (43, 44).

In conclusion, we found no evidence to suggest that a nutrient-enriched formula increased the mean rate of bodyweight gain in AGA late preterm infants from enrolment to 120 days' CA. There is preliminary evidence that suggests the NEF and STF groups differed on some secondary outcomes, including a reduction in infectious illness, though these should be interpreted with caution due to the small sample size and lack of control for multiple testing. Further randomized controlled trials with adequate sample size are warranted to determine if enriching early nutrition can optimize long-term growth and development of this understudied population.

## Data Availability

Sequencing data presented in this study is publicly available from Sequence Read Archive (SRA) NCBI under the Bioproject accession number PRJNA864095.
