# Supplementary material for: Growth of late preterm infants fed nutrient-enriched formula to 120 days corrected age—A randomized controlled trial
Source: Front Pediatr. 2023 May 2;11:1146089. doi: 10.3389/fped.2023.1146089 (PMC10185835; doi:10.3389/fped.2023.1146089)
Supplement: Supplementary file 1 [file Table1.docx]

**Growth of late preterm infants fed nutrient-enriched formula to 120 days corrected age – a randomized controlled trial.**

# Table - Content of Selected Nutrients in the NEF and STF per 100 kcal.

| Nutrient | NEF^a^ | STF |
| --- | --- | --- |
| Calories, per 30ml | 22 | 20 |
| Protein, g | 2.8 | 2.1 |
| Whey: casein ratio | 80:20 | 60:40 |
| Fat, g^b,c^ | 5.3 | 5.3 |
| - Linoleic acid, mg | 86 | 80 |
| - Arachidonic Acid, mg | 25 | 34 |
| - Docosahexaenoic acid | 17 | 17 |
| Carbohydrate, g | 10.4 | 10.9 |
| MFGM, g/L | 5 | - |
| Vitamin A, IU | 450 | 300 |
| Vitamin C, mg | 16 | 12 |
| Vitamin D, IU | 75 | 60 |
| Vitamin E, IU | 4 | 2 |
| Vitamin K, mcg | 9 | 9 |
| Niacin, mcg | 1000 | 1000 |
| Folic Acid, mcg | 26 | 16 |
| Panthothenic acid, mcg | 850 | 500 |
| Biotin, mg | 6 | 3 |
| Choline, mg | 24 | 24 |
| Inositol, mg | 24 | 6 |
| Calcium, mg | 120 | 78 |
| Phosphorus, mg | 66 | 43 |
| Magnesium, mg | 8 | 8 |
| Iron, mg | 1.8 | 1.8 |
| Zinc, mg | 1 | 1 |
| Manganese, mcg | 15 | 15 |
| Copper, mcg | 90 | 75 |
| Iodine, mcg | 21 | 15 |
| Selenium, mcg | 2.8 | 2.8 |
| Sodium, mg | 37 | 27 |
| Potassium, mg | 105 | 108 |
| Chloride, mg | 78 | 63 |

^a^ NEF had whey protein-lipid concentrate (5 g/L, source of bMFGM; Lacprodan® MFGM-10, Arla Foods Ingredients P/S, Denmark)

^b^ Source of ARA was fungal-derived single cell oil and source of DHA was algal-derived single cell oil

^c^ Butyrate: NEF, 15 mg/100 kcal which falls within the range of human milk; STF, None added.

# Fecal Microbiota Composition Additional Methods Information

Samples were returned at ambient temperature directly to the Flinders Medical Center, South Australia, and stored at -80°C prior to microbiome analysis. Fecal DNA was extracted from 2mL of fecal slurry using the DNeasy PowerLyzer PowerSoil kit (QIAGEN, Hidden, Germany), and 16S rRNA amplicon sequencing of the V4 hypervariable region was performed on a MiSeq Reagent Kit v3 (2 x 300bp) (Illumina, San Diego, USA) as described previously.^(20)^ 16S rRNA gene sequencing was performed at the South Australian Genomics Centre, Australia. Paired-end sequencing reads were quality-filtered and analyzed using the QIIME 2 bioinformatics platform,^(45)^ and taxonomy assignment of amplicon sequence variants (ASVs) were performed based on the SILVA rRNA reference database (version 132).^(46)^ Bacterial load in fecal samples were quantitated using a SYBR-based reaction targeting the 16S rRNA gene as described previously.^(47)^ All samples were normalized to a depth of 3,653 sequence reads for alpha diversity (microbial richness and diversity) and microbiota composition analysis.^(47)^ Raw sequence data is publicly available from the Sequence Read Accession database.

**Additional Results**

Differences in fecal microbiota composition at 60 days of age can be attributed to significant differences in the relative abundance of 28 and 6 bacterial taxa in the NEF and STF groups, respectively, compared to the BFR group (Online Figure 2). Of these, 16 and 3 of the bacterial taxa in the NEF and STF groups, respectively, remained consistently altered at 120 days of age. Differentially abundant taxa were predominantly higher in relative abundance in the NEF and STF groups compared to the BFR group, with the exception of *Staphylococcus* and *Cutibacterium,* which were higher in the BFR group at 60 days of age. These differences were no longer present at 120 days of age (Online Figure 2).

# Figure - Fecal microbiome characteristics of nutrient enriched formula, standard term formula and breastfeeding reference infants.

**
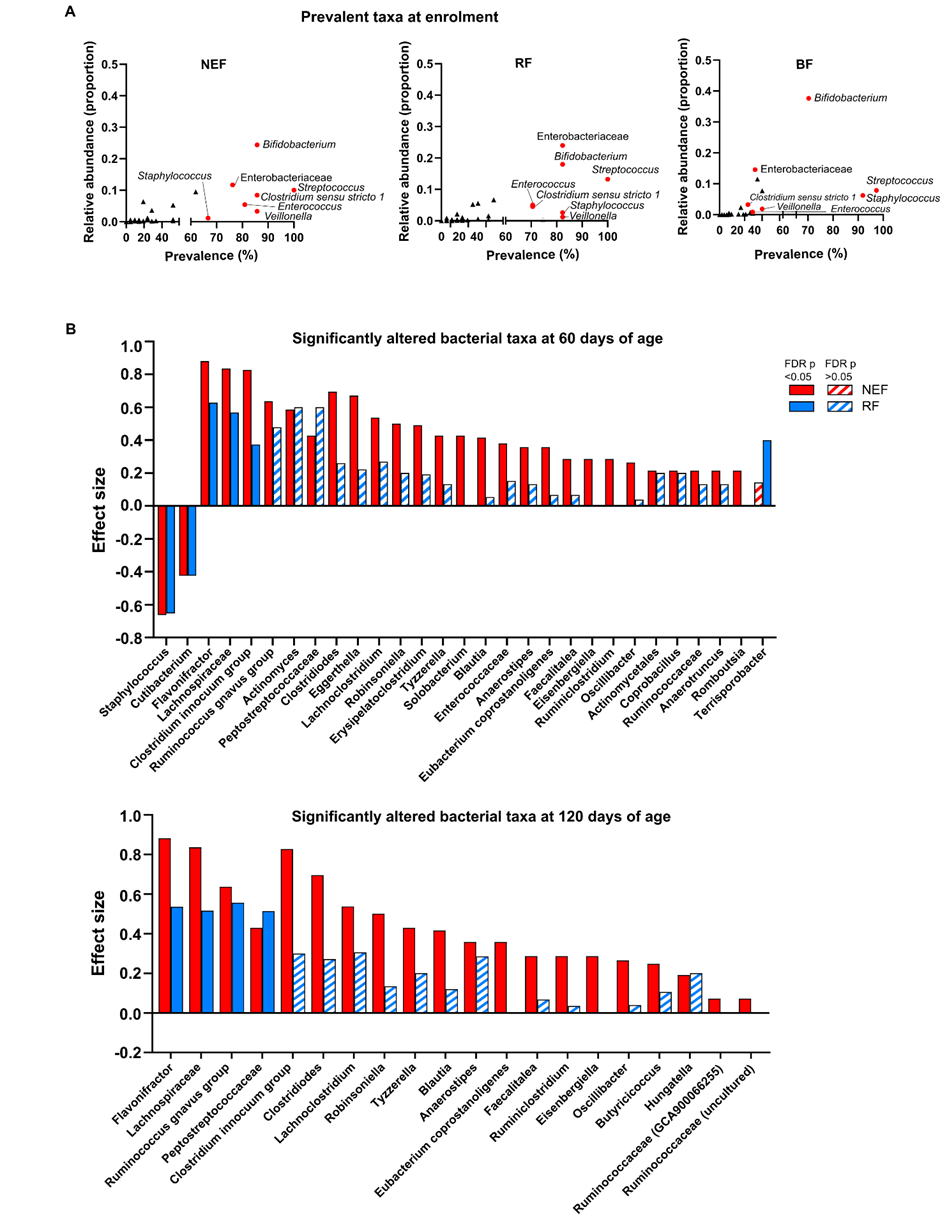
**

(A) Prevalent taxa in nutrient-enriched formula (NEF), routine formula (STF) or reference breastfed (BFR) groups at enrolment. Labelled bacterial taxa are present in >70% infants in either group (red circles). (B) Effect size of bacterial taxa that were differentially abundant in the NEF or STF group compared to the reference BFR group at 60 and 120 days of age. Effect size calculated based on Cliff’s Delta.
